# The generation of metabolic changes for the production of high-purity zeaxanthin mediated by CRISPR-Cas9 in *Chlamydomonas reinhardtii*

**DOI:** 10.1186/s12934-020-01480-4

**Published:** 2020-11-30

**Authors:** Inhwa Song, Jongrae Kim, Kwangryul Baek, Young Choi, ByongCheol Shin, EonSeon Jin

**Affiliations:** 1 Department of Life Science, Research Institute for Natural Sciences, Hanyang University, 222, Wangsimni-ro, Seongdong-gu, Seoul 04763 Republic of Korea; 2 Arca Eir, C-323, Daedeok Biz Center, 17 Techno4-ro, Yuseong-gu, Daejeon, 34013 Republic of Korea

**Keywords:** Zeaxanthin production, Retinal pigment, CRISPR-Cas9, Lycopene epsilon cyclase, *Chlamydomonas reinhardtii*

## Abstract

**Background:**

Zeaxanthin, a major xanthophyll pigment, has a significant role as a retinal pigment and antioxidant. Because zeaxanthin helps to prevent age-related macular degeneration, its commercial use in personalized nutritional and pharmaceutical applications has expanded. To meet the quantitative requirements for personalized treatment and pharmaceutical applications, it is necessary to produce highly purified zeaxanthin.

**Results:**

In this study, to meet the quantitative requirements for industrial applications, we generated a double knockout mutant which is gene-edited by the CRISPR-Cas9 ribonucleoprotein-mediated knock-in system. The lycopene epsilon cyclase (*LCYE*) was edited to the elimination of α-branch of xanthophyll biosynthesis in a knockout mutant of the zeaxanthin epoxidase gene (*ZEP*). The double knockout mutant (*dzl*) had a 60% higher zeaxanthin yield (5.24 mg L^− 1^) and content (7.28 mg g^− 1^) than that of the parental line after 3 days of cultivation. Furthermore, medium optimization improved the 3-day yield of zeaxanthin from the *dzl* mutant to 6.84 mg L^− 1^.

**Conclusions:**

A *Chlamydomonas* strain with the elimination of lutein production by gene editing using CRISPR-Cas9 has been successfully developed. This research presents a solution to overcome the difficulties of the downstream-process for the production of high-purity zeaxanthin.

## Background

As the major carotenoids, xanthophyll pigments absorb energy at wavelengths ranging between 400 and 530 nm, transmit this energy to the reaction center of the photosystem, establish and maintain the structure of chloroplasts [1–3]. Zeaxanthin is one of the oxygen-containing xanthophyll pigments synthesized from β-carotene in most photosynthetic organisms. Under high-light conditions, de-epoxidation of violaxanthin is induced by the pH difference across the thylakoid membrane, converting violaxanthin to zeaxanthin [1, 4]. In several organisms zeaxanthin has been reported to have a significant role in the non-photochemical quenching mechanism that dissipates the excess energy of excited chlorophyll *a* as heat [4, 5]. Like in plant, zeaxanthin is also found in the animal retina, predominantly in the central fovea [6–8] and exists in two isoforms, zeaxanthin and meso-zeaxanthin [9]. They filter blue light and protects photoreceptors against photo-oxidative stress in retina [10, 11]. In connection with these biological functions, several studies have demonstrated that zeaxanthin is associated with the enhancement of visual performance [12, 13] and prevention of age-related macular degeneration [14, 15]. Consequently, the commercial value of zeaxanthin has expanded from animal feed to nutritional supplements and pharmaceutical preparations. In recent years, with the increase in the market, the importance of purification and quantification of zeaxanthin has emerged. It was noted that the amounts of zeaxanthin and lutein in supplements should be balanced to effectively increase macular pigment optical density [16] and that the intake needed by different individuals differs, depending on genetic and metabolic factors [11]. To meet the quantitative requirements for personalized treatment and pharmaceutical applications, it is necessary to produce highly purified zeaxanthin.

*Chlamydomonas reinhardtii* has been studied as a green microalgal model to elucidate cellular mechanisms such as photosynthesis, gene expression, and pigment biosynthesis [17, 18]. There are many laboratory strains of *C. reinhardtii* with unique characteristics; CC-4349 is the best host for pigment production, showing high growth rate and carotenoid productivity [19]. Due to deficiencies in its cell wall, it is easy to improve the strain’s abilities via genetic engineering using transformation [20]. Using targeted mutagenesis by DNA-free CRISPR-Cas9 ribonucleoprotein (RNP), we have generated knockout mutants of the zeaxanthin epoxidase gene [21]. In these *ZEP* mutants, constitutive accumulation of zeaxanthin and lutein was achieved. Although the possibility of commercial application of *ZEP* mutant was confirmed, purification of zeaxanthin from it was difficult because of the simultaneous production of lutein; lutein has a structure of (3R,3′R,6′R)-β,ε-carotene-3,3′-diol which is similar with zeaxanthin structure of (3R,3′R)-β,β-carotene-3,3′-diol [8]. In addition, since α-carotene and β-carotene, which are precursors of lutein and zeaxanthin respectively, branch out from lycopene, competition for tetraterpene from the upstream pathway is inevitable [22]. Therefore, specific production of zeaxanthin is required for high productivity and effective purification.

In this study, we generated double gene knockout mutants of *C. reinhardtii* using a CRISPR-Cas9 RNP-mediated knock-in system. We targeted the lycopene epsilon cyclase encoding gene (*LCYE*) using the *ZEP* mutant as a parental line to inhibit the biosynthesis of α-carotene and generated several *ZEP*/*LCY*E double knockout mutants. We selected the best dKO strain for specific production of zeaxanthin based on growth rate and pigment productivity and compared zeaxanthin content and yield of this mutant and the parental strains under mixotrophic growth conditions. To increase zeaxanthin productivity, we adjusted the concentrations of medium components essential for cell growth and improved cell density and biomass.

## Results and discussion

### Generation of double knockout mutants of *C. reinhardtii*

**Fig. 1:**
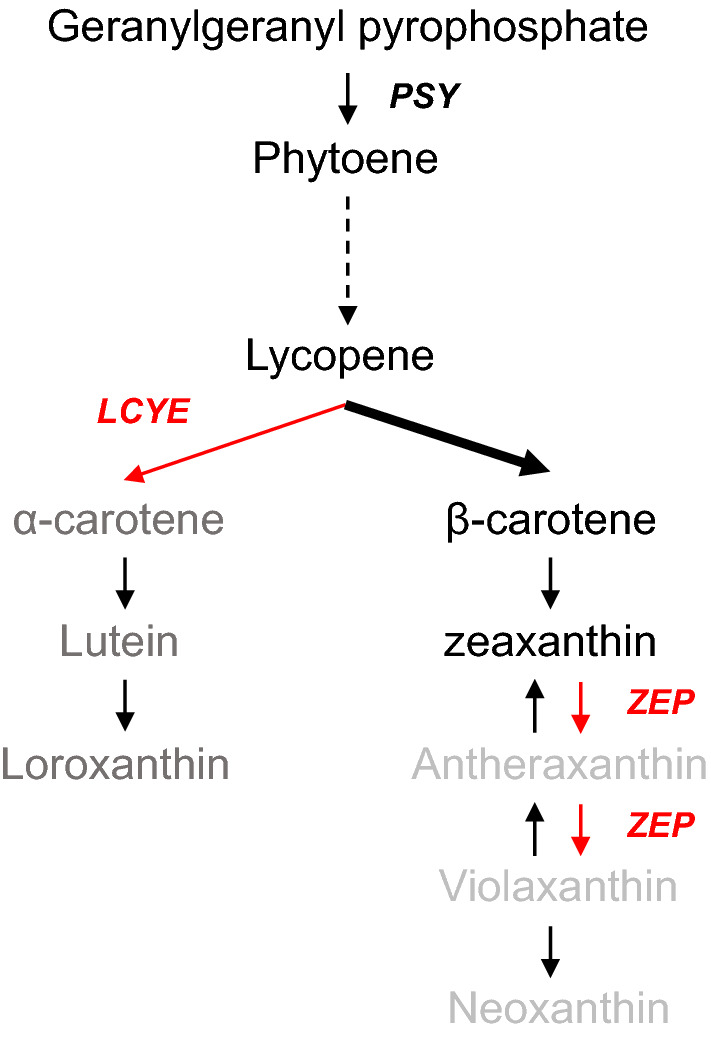
Schematic diagram of the carotenoid biosynthesis pathway in *C. reinhardtii dzl* mutant. Dashed arrow implies intermediate enzymes and products. The enzymes in red were targeted by CRISPR-Cas9 RNP mutagenesis. The grey or light grey-colored pigments were removed in the *LCYE* or *ZEP* knockout mutants, respectively. The bold arrow indicates that lycopene flux is concentrated in the *dzl*

*C. reinhardtii* has been used as a model organism to study carotenoid synthesis and the pathways involved are well understood (Fig. 1). We previously generated single *ZEP* knockout mutants of CRISPR-Cas9 RNP-mediated knockout without using the hygromycin resistance gene as a selective marker in *C. reinhardtii* CC-4349 [21]. The absence of zeaxanthin epoxidase enabled zeaxanthin accumulation without high light induction, however, because of lutein production, it was difficult to efficiently purify zeaxanthin. In this study, to facilitate production and the purification of zeaxanthin, we used CRISPR-Cas9 to disrupt the *LCYE* gene, which is involved in α-cyclization of lycopene, in the *ZEP* mutant as a parental line and generated *ZEP* and *LCYE* double knockout (*dzl*) mutants.

**Fig. 2:**
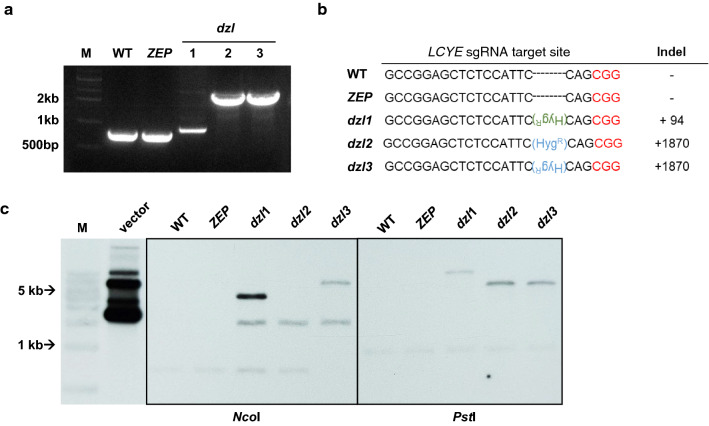
Characterization of *dzl* mutants. **a** Genomic PCR of the *LCYE* gene in the wild type and the *ZEP*, and *dzl* mutants. The intact *LCYE* gene yield a 752-bp PCR fragment. **b** DNA sequence alignment at the *LCYE* locus of the wild type, *ZEP*, and the *dzl* mutants. The 20-bp sequence before the PAM sequence (red) was used for in vitro sgRNA transcription. The inserted *aph7* gene is shown in blue (full-length) and green (partial). Upside-down characters represent insertions in the reverse orientation. The right column indicates the length of the insertions at the target locus. **c** Southern blot analysis of *dzl* mutants. Genomic DNA (20 μg) of each strain was digested with either *Nco*I or *Pst*I and probed with a PCR fragment corresponding to the *aph7* gene. The pChlamy3 vector was used as a positive control

For target-specific mutagenesis, four sgRNA sequences were selected from the first exon of the *LYCE* gene. Each in vitro assembled CRISPR-Cas9 RNP combination was co-transformed with the *aph7* gene, which is used for the easy selection by CRISPR-Cas9-mediated knock-in strategy [23]. Among the four target sites, the fourth sgRNA showed high cleavage efficiency and produced three *dzl* mutants with different insertions (Fig. 2). According to Sanger sequencing, in all three mutants, the cleavage occurred between the third and fourth base pairs before the PAM sequence, resulting in insertions in the *LCYE* gene (Fig. 2b). *dzl1* had a 94-bp insertion of the partial sequence (specifically, 3′ UTR region) of the *aph7* gene, and the other two mutants had 1870-bp insertion of full-length aph7 gene in either forward or reverse directions. The *aph7* gene used for the knock-in at the target site does not exclude the possibility of random integrations at a different location, which might cause the unexpected side effects of the undesired mutant phenotype. Therefore, an additional Southern blot analysis was carried out to assess the number of insertion events in the mutant genome. In Fig. 2c, Southern blot analysis revealed that *dzl1* and *dzl3* had more than two copies and *dzl2* might have only one copy of the *aph7* gene in the genome. Therefore, we further investigated the effect of the random integrated extra *aph7* gene in the mutant genome on the pigment profiles and growth behaviors.

**Fig. 3:**
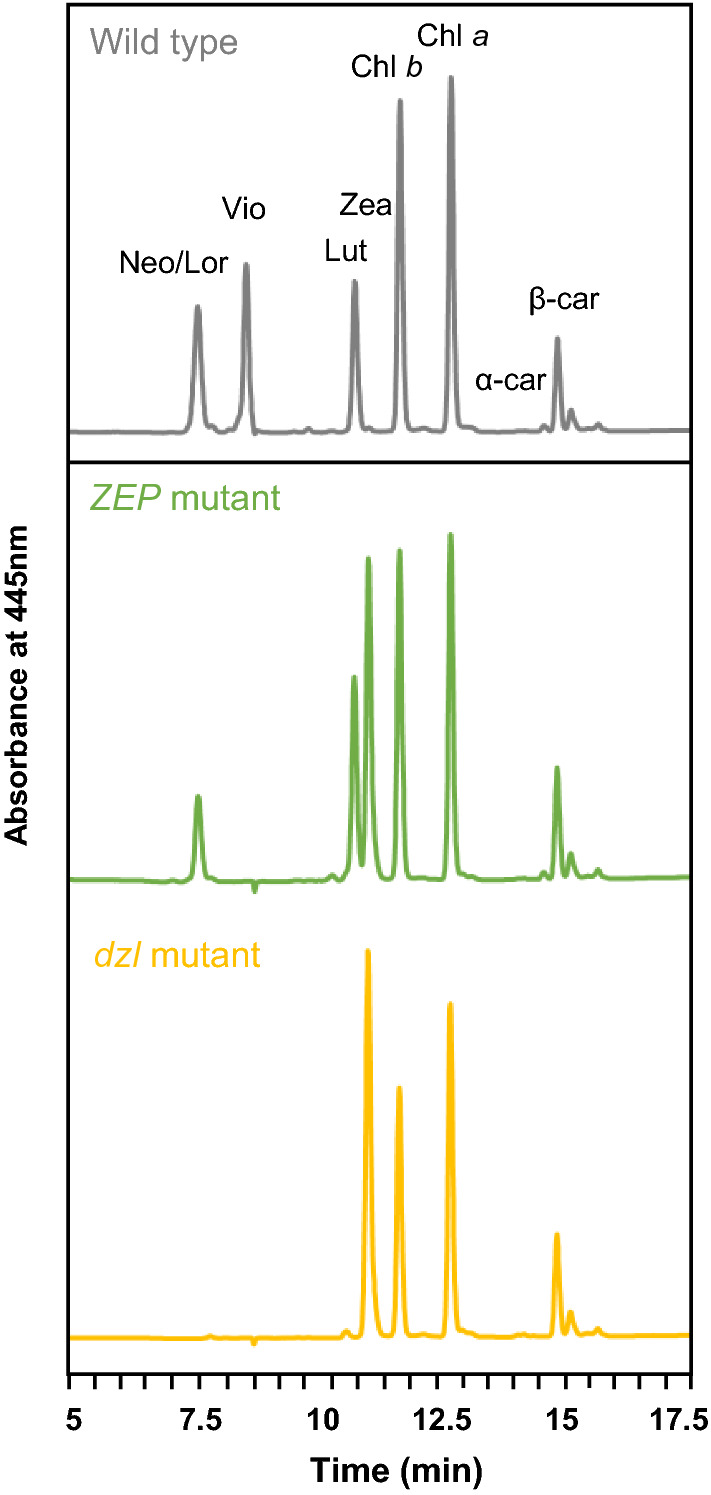
Pigment profiles. Pigments were extracted with 90% acetone from the wild type, *ZEP*, and *dzl* cultured under 60 μmol photons m^–2^ s^–1^. Neo, neoxanthin; Lor, loroxanthin; Vio, violaxanthin; Lut, lutein; Zea, zeaxanthin; Chl b, Chlorophyll *b*; Chl a, Chlorophyll *a*; α-car, α-carotene; β-car, β-carotene

**Fig. 4:**
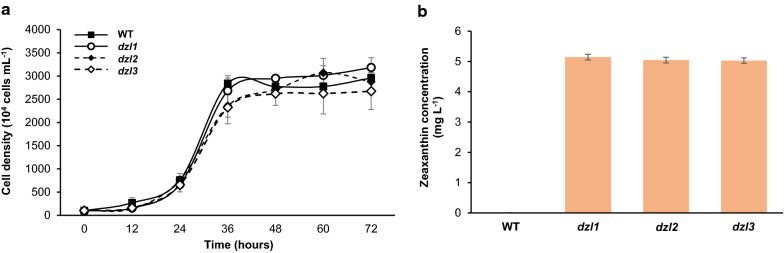
Growth analysis and measurement of zeaxanthin content of *dzl* mutants (**a**) Growth of wild type and *dzl* mutants cultured in TAP media at 25 °C under 60 μmol photons m^–2^ s^–1^. **b** Zeaxanthin yield extracted from each strain at 60 h during cultivation. Values represent the mean and standard error of three independent experiments

To select the best strain for zeaxanthin production, we compared the growth and zeaxanthin productivity of the three *dzl* mutants. Because the expression of *LCYE* was inhibited, the *dzl* mutant could not synthesize carotenoids of the α-branch, which were intact in the *ZEP* mutant. It was shown that the gradual changes of pigment profiles caused by the sequential CRISPR-Cas9 knockout in Fig. 3. As predicted from the carotenoid synthesis pathway, the *ZEP* mutant had the peaks of lutein and zeaxanthin, whereas the *dzl* mutant had zeaxanthin as the main carotenoid pigment. All of them grew slowly compared to the wild type during the exponential phase, but all mutants had similar cell numbers with the wild type at the end of growth (Fig. 4a). Under low-light conditions, the wild type accumulated no detectable zeaxanthin, whereas all *dzl* mutants produced over 5 mg L^–1^ zeaxanthin (Fig. 4b). Because *dzl*1 showed the highest cell density and zeaxanthin yield, it was selected as the optimal zeaxanthin production strain and used in all subsequent experiments under the name of *dzl*.

### Zeaxanthin production in double knockout mutant

**Fig. 5:**
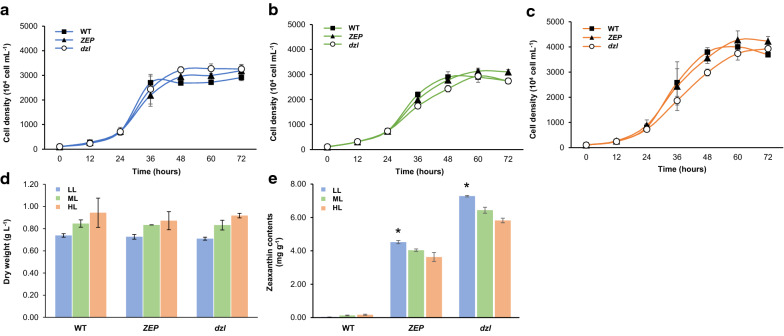
Effects of light intensity on growth and zeaxanthin production in zeaxanthin-accumulating mutants of *C. reinhardtii.* **a**–**c** Growth of the wild type, *ZEP*, and *dzl* cultured in TAP media at 25 °C under (**a**) low light (60 μmol photons m^–2^ s^–1^), **b** moderate light (200 μmol photons m^–2^ s^–1^), or (**c**) high light (400 μmol photons m^–2^ s^–1^). The initial cell concentration was 10^6^ cells mL^−1^. Values represent the mean and standard error of two independent experiments. **d** Biomass production under three different light of each strain at 72 h. **e** Zeaxanthin content extracted from each strain at 72 h. Statistical analyses were performed using Student’s t-test, * p < 0.05. LL, low light; ML, moderate light; HL, high light

We compared the growth of the wild type and zeaxanthin-accumulating mutant what we selected above (*dzl*) in three different light conditions (Fig. 5). Under all light conditions, the mutant and the WT had a similar final cell density in the stationary phase (Fig. 5a–c). However, a slight reduction in the growth rate of dzl was observed during the exponential growth phase under high light condition (Fig. 5c). The absence of lutein, violaxanthin, and neoxanthin is thought to affect the dzl growth rate, especially under high light growth condition. Additionally, comparing the biomass productivity, there was no difference in cell density at stationary phase between low and moderate light conditions, however the biomass of cells cultivated under moderate light was increased by 15–17% compared to that of cells cultivated under low light (Fig. 5d). High light cultivation increased the number of all the cells with a 20–30% increased biomass compared to low light cultivation. However, since zeaxanthin yield did not change in the mutants under higher light conditions despite the increase in biomass or cell density, the highest zeaxanthin yield was achieved under low light (Fig. 5e).

**Fig. 6:**
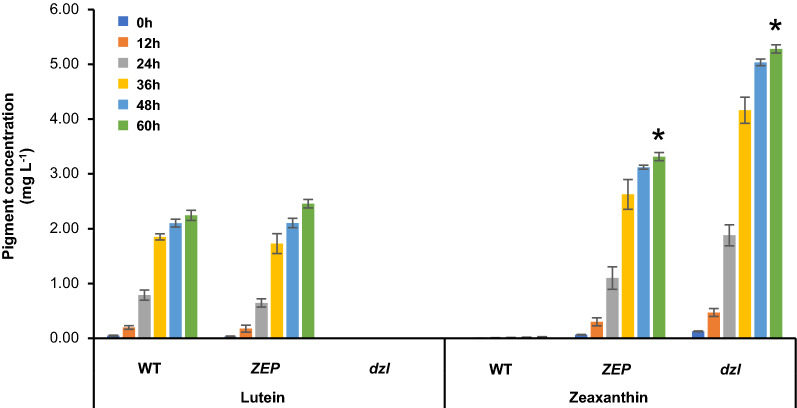
Pigment productivity from the zeaxanthin-accumulating mutants under mixotrophic conditions. Time course of lutein and zeaxanthin concentrations. Cells were inoculated with the initial cell concentration of 10^6^ cells mL^–1^. Statistical analyses were performed using Student’s t-test, * p < 0.05. Values are the mean and standard error of three independent experiments

To maximize the zeaxanthin content and avoid the use of excessive light energy, we compared zeaxanthin productivity from these strains cultured under low light. Pigment production increased with cell growth and *ZEP* simultaneously produced lutein and zeaxanthin, while *dzl* produced only zeaxanthin at higher concentrations (Fig. 6). In conclusion, owing to a double knockout of *ZEP* and *LCYE*, metabolic flux was concentrated into the β-carotene biosynthesis pathway from the lycopene branch point and the increased β-carotene resulted in the production of zeaxanthin but not lutein. *dzl* had a zeaxanthin content of 7.28 mg g^–1^, about 60% higher than that of *ZEP* (4.56 mg g^–1^), and a zeaxanthin yield of 5.24 mg L^–1^, also about 60% higher than that of *ZEP* (3.31 mg L^–1^).

### Enhancement of zeaxanthin production

**Fig. 7:**
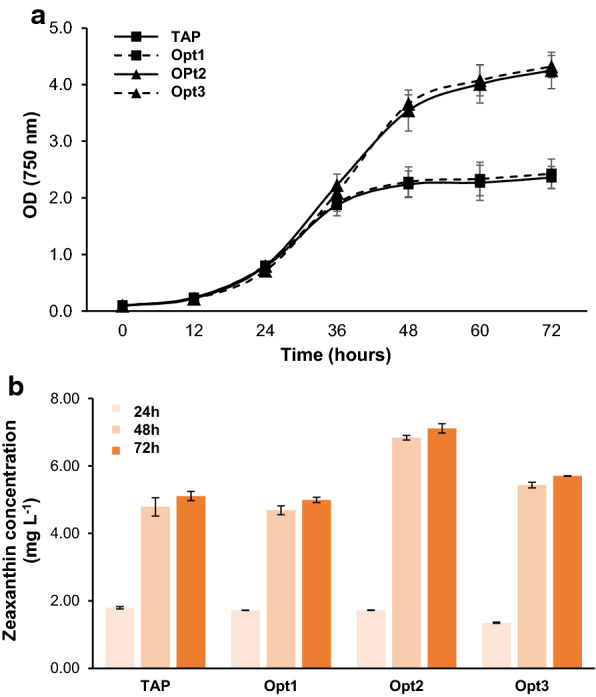
Medium optimization to increase zeaxanthin production from *dzl.* **a** *dzl* was grown in TAP media or three optimized media with adjusted concentrations of the medium components at 25 °C under low light. The absorbance of culture at 750 nm was measured. **b** Time course of zeaxanthin concentration in cells grown as in (**a**) and harvested every 24 h

Blocking the pathway competing with zeaxanthin biosynthesis increased the zeaxanthin pool from a given amount of carotenoids in *dzl*. However, since it has a limit to increase the total pool of carotenoids within the cell, we attempted medium optimization to increase zeaxanthin productivity. We compared growth in four TAP media with adjusted concentrations of nitrogen, phosphate, or acetic acid, which are essential for cell growth (Fig. 7a). Moderate reduction of nitrogen and phosphorus concentrations in Opt1 did not affect cell growth and pigment production. The increased acetic acid in both Opt2 and Opt3 promoted cell growth after 48 h; however, in Opt3, which had nitrogen and phosphorus concentrations reduced, pigment concentration was lower than that in Opt2 (Fig. 7b). Therefore, we cultured *dzl* in Opt2 for high zeaxanthin production and could enhance the biomass and zeaxanthin production of *dzl* compared to those in TAP media (Table 1). Interestingly, the carbon source from photosynthesis under high-light conditions did not increase zeaxanthin production, but acetate as a carbon source did increase zeaxanthin in Opt2. It seems that the increase in the levels of metabolites from acetate assimilation acted upstream of carotenoid synthesis in cells specifically adapted to mixotrophic conditions. As a result, zeaxanthin yield after the 3-day cultivation increased from 5.24 to 6.84 mg L^–1^ (Table 1).

**Table. 1:** Zeaxanthin productivity of *dzl* cultured in TAP media or in Opt2 media

	Yield (mg L^−1^)		Biomass (g L^−1^)	Content (mg g^−1^ DCW)	
	Lutein	Zeaxanthin		Lutein	Zeaxanthin
TAP	N.D^b^	5.24 ± 0.03	0.71 ± 0.02	N.D^b^	7.28 ± 0.04
Opt2	N.D^b^	6.84 ± 0.20^a^	1.27 ± 0.04^a^	N.D^b^	5.40 ± 0.16

^a^Statistical difference between TAP vs. Opt2 condition (p < 0.05)

^b^N.D, not detectable; DCW, dry cell weight

Due to the important role of zeaxanthin in photosynthesis, research on zeaxanthin has been conducted in many organisms. Several zeaxanthin-producing mutants with phenotypes similar to that of *dzl* have been isolated by random mutagenesis, including in *Arabidopsis thaliana*, *Scenedesmus obliquus*, and *C. reinhardtii* [2, 24, 25]. These studies determined the physiological effects of zeaxanthin accumulation on photosynthesis including photosynthetic capacity, non-photochemical quenching, and photosynthetic apparatus organization, and confirmed that the absence of xanthophylls other than zeaxanthin does not affect cell survival and growth. The physiological evidence from these studies showed the possibility of zeaxanthin production as the single xanthophyll; however, they did not focus on that as such. Whereas our *dzl* mutant was generated as a specialized strain to produce high-purity zeaxanthin. When cultured in optimized media mixotrophically, *dzl* produced 30% more zeaxanthin with increased cell density and biomass in comparison with standard TAP media.

### Further application of metabolic engineering to the *dzl*

To date, zeaxanthin-accumulating microorganisms have been isolated from nature or generated by random mutagenesis to overcome the limits of yield and high production cost from land plants, the major zeaxanthin producers [26]. Of the reported zeaxanthin-accumulating microalgae, wild-type *Chlorella saccharophila* and the *bkt1* mutant of *Chlorella zofingiensis* have the highest zeaxanthin content, 11.2 mg g^–1^ DCW and 7 mg g^–1^ DCW, respectively [27, 28]. Both *Chlorella* strains are fast-growing microalgae with high biomass yield and carotenoid levels, making them good hosts for zeaxanthin production. However, it is hard to extract the pigments from *Chlorella* because of its thick cell wall. Several extraction methods have been attempted, but those are laborious and complete extraction is difficult [29, 30]. In addition, since the *LCYE* gene is intact in both strains, lutein is produced along with zeaxanthin and could interfere with zeaxanthin purification. On the other hand, as the first microalgal mutant generated by targeted mutagenesis to enable the specific production of zeaxanthin, *dzl* is highly efficient in terms of extraction and purification of pigments. The background strain CC-4349 of *dzl* has a no cell wall, grows quickly to saturation and pigment extraction from it is easily processed with 90% acetone without cell disruption by physical means. The absence of lutein in *dzl* is also advantageous for zeaxanthin purification because it obviates the need to increase chromatographic resolution and the conventional pigment separation method can be used. Thus, *dzl* is a good candidate for production and purification of zeaxanthin.

Metabolic engineering strategies for overexpression of native or heterologous genes in yeast, bacteria, and cyanobacteria have been successfully applied, resulting in high yield of zeaxanthin [26]. To compete with zeaxanthin producing bacteria and *Chlorella* and enable the commercial use of *dzl* for zeaxanthin production, metabolic flux should be regulated in addition to the knockout of the *LCYE* gene to increase the total pool of carotenoids within the cell. Since metabolic engineering of *C. reinhardtii* is advantageous due to the already known native pigment biosynthesis pathway and easy transformation, *dzl* is an ideal candidate for further strain improvement. The carotenoid biosynthesis pathway is well conserved between plants to microalgae, and the rate-limiting enzymes have been identified. In *Arabidopsis*, overexpression of *DXS* and *DXR*, the rate-limiting enzymes that regulate the isoprenoid flux of the methylerythritol 4-phosphate (MEP) pathway, has shown to increase carotenoid productivity [31, 32]. In *Chlamydomonas*, overexpression of *PSY,* considered a key enzyme in carotenoid biosynthesis (Fig. 1), increased the production of lutein and violaxanthin [33]. Therefore, overexpressing these genes as well as co-expression of *CHYB* or *CYP97A5/6* which converts from β-carotene to zeaxanthin in *dzl* should be attempted to increase cellular carbon flux to the carotenoid pathway and to further increase zeaxanthin production eventually.

## Conclusion

In this study, we characterized the first microalgal mutants producing zeaxanthin as a sole xanthophyll generated by CRISPR-Cas9 RNP-mediated mutagenesis. The introduced mutation increased the zeaxanthin content to 7.28 mg g^–1^ by removing α-carotene biosynthesis and pigment extraction and purification from this mutant can be easily achieved using conventional chromatography methods. Through medium optimization, a zeaxanthin yield of 6.84 mg L^−1^ was obtained after 3-days of cultivation. Also, further metabolic engineering could enhance the productivity. The production of highly purified zeaxanthin from this mutant could provide sufficient amounts of zeaxanthin for personalized treatment and pharmaceutical applications.

## Methods

### Algal strain and culture conditions

*Chlamydomonas reinhardtii* CC-4349 cw15 mt- and mutant strains were maintained in Tris–acetate phosphate (TAP) medium (7.5 mM NH_4_Cl, 0.62 mM K_2_HPO_4_, 0.41 mM KH_2_PO_4_, and 1 mL L^–1^ glacial acetic acid). Cells were cultured mixotrophically on an orbital shaker at 120 rpm under continuous white fluorescence light (60 μmol photons m^–2^ s^–1^) at 25 °C. For the cultivation on higher light conditions, cells were cultured under moderate light (200 μmol photons m^–2^ s^–1^) and high light (400 μmol photons m^–2^ s^–1^). Initially, cells (10^6^ cells mL^–1^) was inoculated into 50 mL media in 250 mL flasks. For medium optimization, the concentrations of nutrients were adjusted (Opt1: 5.625 mM NH_4_Cl, 0.465 mM K_2_HPO_4_, 0.3075 mM KH_2_PO_4_, Opt2: 2 mL L^–1^ glacial acetic acid, Opt3: 5.625 mM NH_4_Cl, 0.465 mM K_2_HPO_4_, 0.3075 mM KH_2_PO_4_, 2 mL L^–1^ glacial acetic acid).

### CRISPR-Cas9 RNP-mediated knock-in

*Chlamydomonas* transformation was performed as described previously with a few modifications [21, 23]. Briefly, 100 μg lyophilized Cas9 protein (ToolGen, Seoul, South Korea) dissolved in nuclease-free water with 50% glycerol and in vitro transcribed 70 μg sgRNA were premixed for 10 min at room temperature to form each RNP complex. An *aph7* gene, which confers the hygromycin resistance, was prepared by PCR amplification from the pChlamy3 vector with a specific primer set (F: 5′-ATG ATT CCG CTC CGT GTA AAT G-3′, R: 5′-AGT ACC ATC AAC TGA CGT TAC ATT C-3′). Then, 500 × 10^4^ cells were incubated with the RNP complex and 1 μg of *aph7* for 5 min and transformed with a Gene Pulser Xcell Electroporation System (Bio-Rad, CA, USA) according to the recommended protocol from the GeneArt *Chlamydomonas* Engineering Kit (Life Technologies, CA, USA). After electroporation, cells were incubated in TAP media supplemented with 40 mM sucrose in 6-well plates for 24 h. Cells were harvested and plated on TAP media containing 1.5% agar with 25 μg mL^–1^ of hygromycin-B (Life Technologies, CA, USA) for mutant selection.

### Mutant screening and genotypic characterization

Colonies from TAP agar plates were transferred to 96-well plates, and the individual cells were incubated in TAP media with hygromycin-B for 3 days. Transformed cells were subjected to colony PCR with specific primers adjacent to sgRNA target sites in *LCYE* (F: 5′-TGG TGA AAT CTA GCG TCG GCT-3′, R: 5′-GAC GCA ATT GCC GCT TGA GA-3′) for mutant screening. Knockout mutants with the *aph7* DNA inserted were selected, their genomic DNA was isolated, and the target region was PCR-amplified for sequence confirmation. The PCR products were separated on an agarose gel and sequenced using the Sanger method (Macrogen, Seoul, South Korea).

### Southern blot analysis

*Chlamydomonas* transformation was performed as described in the manufacturer’s protocol (Gene Images AlkPhos Direct Labeling and Detection System Kit, GE Healthcare, IL, USA). Purified genomic DNA (20 μg) was digested with the *Nco*I and *Pst*I restriction enzymes and the digests were separated on a 0.8% agarose gel and transferred to a positively charged nylon membrane (Amersham Hybond-N^+^, GE healthcare, IL, USA). Probes, the *aph7* gene, obtained by PCR amplification from the pChlamy3 vector were labeled with alkaline phosphatase provided in the kit. The transferred DNA was cross-linked using UV cross-linker and subjected to labeling, hybridization, washing, and signal detection according to the manufacturer's protocol.

### Pigment quantification

Cells were harvested every 12 h and pigments were extracted with 90% (v/v) acetone by pipetting until the cells became colorless. The supernatants were subjected to analysis using a Shimadzu Prominence HPLC model LC-20AD (Shimadzu, Kyoto, Japan) equipped with a Spherisorb 5.0 μm ODS1 4.6 × 250 mm cartridge column (Waters, Milford, USA). Pigment concentrations were calculated from absorbance at 445 nm and 670 nm as described previously [19]. All quantitative analysis were carried out at least in triplicate. Student’s t-test was performed to determine the statistical significance of differences in pigment production. A significant difference indicating by the asterisk was considered at a p value < 0.05.

## Data Availability

All data generated or analyzed in this study are included in this article.
